# Stable tissue-mimicking phantoms for longitudinal multimodality imaging studies that incorporate optical, CT, and MRI contrast

**DOI:** 10.1117/1.JBO.28.4.046006

**Published:** 2023-04-20

**Authors:** Mengyang Zhao, Mingwei Zhou, Xu Cao, Jinchao Feng, Brian W. Pogue, Keith D. Paulsen, Shudong Jiang

**Affiliations:** a Dartmouth College, Thayer School of Engineering, Hanover, New Hampshire, United States; b Beijing University of Technology, Beijing Key Laboratory of Computational Intelligence and Intelligent System, Faculty of Information Technology, Beijing, China

**Keywords:** tissue phantom, optical property, multimodality imaging, spectroscopy

## Abstract

**Significance:**

Tissue phantoms that mimic the optical and radiologic properties of human or animal tissue play an important role in the development, characterization, and evaluation of imaging systems. Phantoms that are easily produced and stable for longitudinal studies are highly desirable.

**Aim:**

A new type of long-lasting phantom was developed with commercially available materials and was assessed for fabrication ease, stability, and optical property control. Magnetic resonance imaging (MRI) and x-ray computed tomography (CT) contrast properties were also evaluated.

**Approach:**

A systematic investigation of relationships between concentrations of skin-like pigments and composite optical properties was conducted to realize optical property phantoms in the red and near-infrared (NIR) wavelength range that also offered contrast for CT and MRI.

**Results:**

Phantom fabrication time was <1  h and did not involve any heating or cooling processes. Changes in optical properties were <2% over a 12-month period. Phantom optical and spectral features were similar to human soft tissue over the red to NIR wavelength ranges. Pigments used in the study also had CT and MRI contrasts for multimodality imaging studies.

**Conclusions:**

The phantoms described here mimic optical properties of soft tissue and are suitable for multimodality imaging studies involving CT or MRI without adding secondary contrast agents.

## 1 Introduction

Tissue phantoms that mimic physical and biochemical characteristics of target human or animal tissue serve an important role in the comparison, evaluation, and quality control of associated imaging systems for clinical diagnostic and treatment surveillance applications.^1^^–^^6^ Past studies on optical phantoms have been focused on mimicking the optical properties of tissue and representing their three-dimensional (3D) structures.^7^^–^^11^ Recent typical examples include the 3D-printed biophotonic phantoms to mimic the cerebrovascular module by filling whole bovine blood to the 3D-printed thin-cylindrical photopolymer channels^12^ and the 3D-printed phantoms with complex layered structures that mimic the brain based on magnetic resonance imaging (MRI) atlas data.^13^ However, these phantoms either are focused on mimicking the optical properties at the particular wavelengths^13^ (due to the optical properties of the printed material) or cannot maintain the optical properties over a long term^12^ (due to the nature of blood). Moreover, creating multimodal phantoms presents challenges in terms of fabrication complexity, structural integrity, shelf life, and optical spectral coverage.^2^^,^^4^^,^^14^ Often multiple contrast agents are needed in the phantoms for multimodality imaging, which increases the complexity of phantom construction. Commonly, CT or MR contrasts are incorporated in an aqueous substrate in optical phantoms, which creates internal boundaries between structures. These materials often diffuse at different rates during fabrication, blurring boundaries, and limiting spatial accuracy and property values.

To overcome these common problems, a new type of phantom has been developed that is easy to fabricate and provides long-term stability. Specifically, a translucent, low viscosity, silicone soft gel was used as the base material, along with pink and white pigments to control the phantom optical properties over the red and near-infrared (NIR) wavelength range. By adjusting the concentrations of the two pigments, optical properties can be tuned to the desired values and wavelength range with spectral features similar to soft tissue. These phantoms can be made in an hour without involving any heating nor cooling processes, and changes in optical properties were shown to be <2% over 12 months. In addition, MRI or CT signal intensities also varied with pigment concentrations, eliminating the need to incorporate secondary contrasts for multimodality studies involving these modalities.

## 2 Methods

### 2.1 Phantom Preparation

**Fig. 1:**
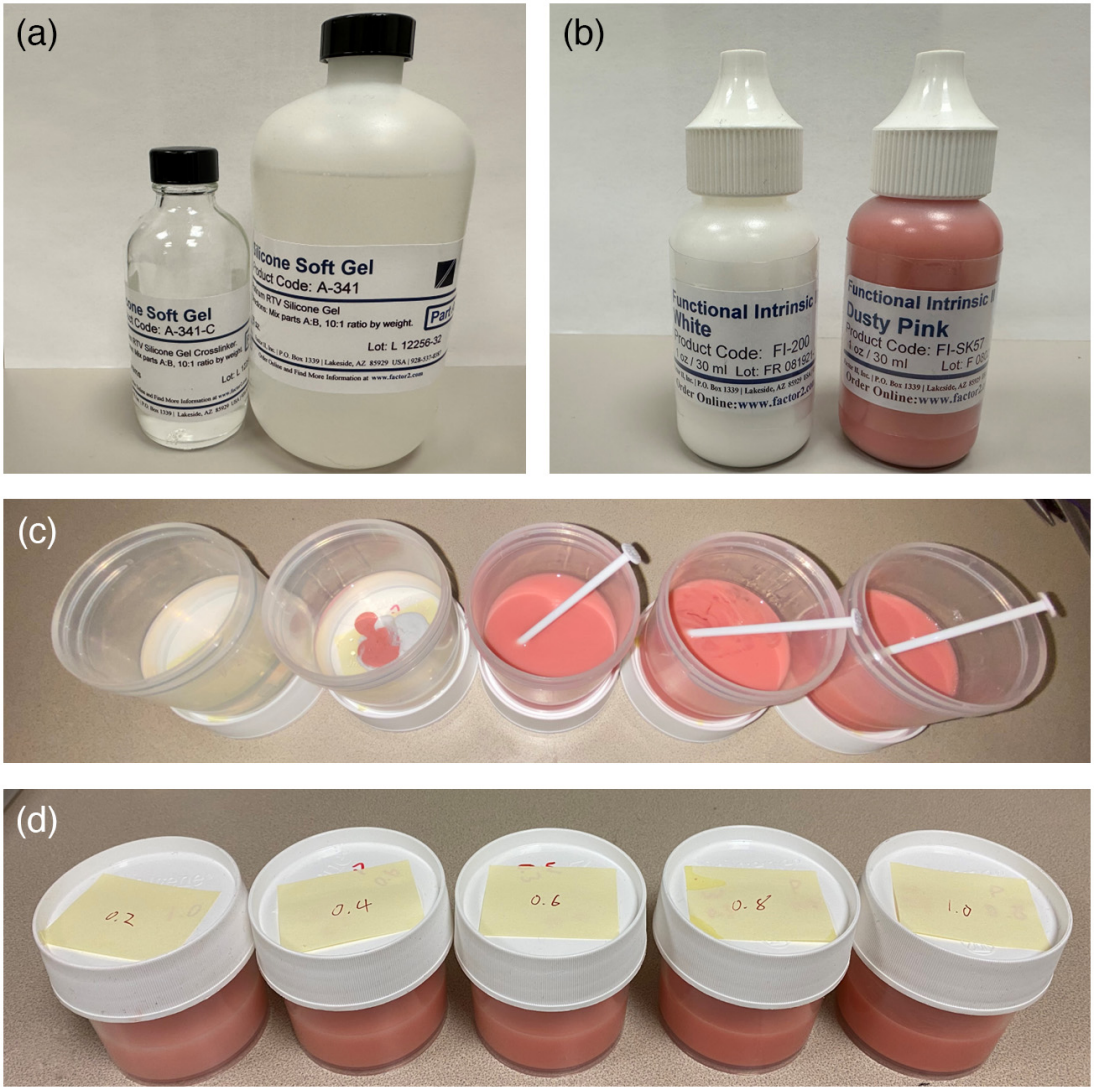
Materials, procedure, and examples of small soft gel phantoms. (a) Silicone soft gel base A and catalyst B (A-341 Factor2). (b) Silicone pigment materials (FI-SK, functional intrinsic skin colors, Factor2). (c), (d) Production of a series of small soft gel phantoms with different optical properties.

Figure 1 shows the materials, procedure, and resulting phantoms having different optical properties. A translucent, low viscosity, silicone soft gel (A-341 Factor2) and catalyst B [Fig. 1(a)] were used as the base material, and pink and white pigments (FI-SK, functional intrinsic skin colors, Factor2, ) [Fig. 1(b)] were added to control optical properties.^15^ To fabricate a phantom, we first mixed n ml of base A with proportional amounts of pigments in a container and stirred for 3 min to ensure their uniformity [Fig. 1(c)].

Then, we added n/10  ml of catalyst B of A-341 into the mixture and stirred the solution for 3 more minutes to allow the catalyst to conjugate with the base, and finally, we poured the mixture into a mold using a funnel. A stir rod was used to mix pigments manually into the base materials, and air bubbles were released naturally during the stirring and curing. After waiting for ∼60  min, the phantom was fully cured without degassing. The total procedure required about 60 min, and all procedures were carried out at room temperature.

### 2.2 3D-Printed Mold for Breast-Shaped Phantoms

**Fig. 2:**
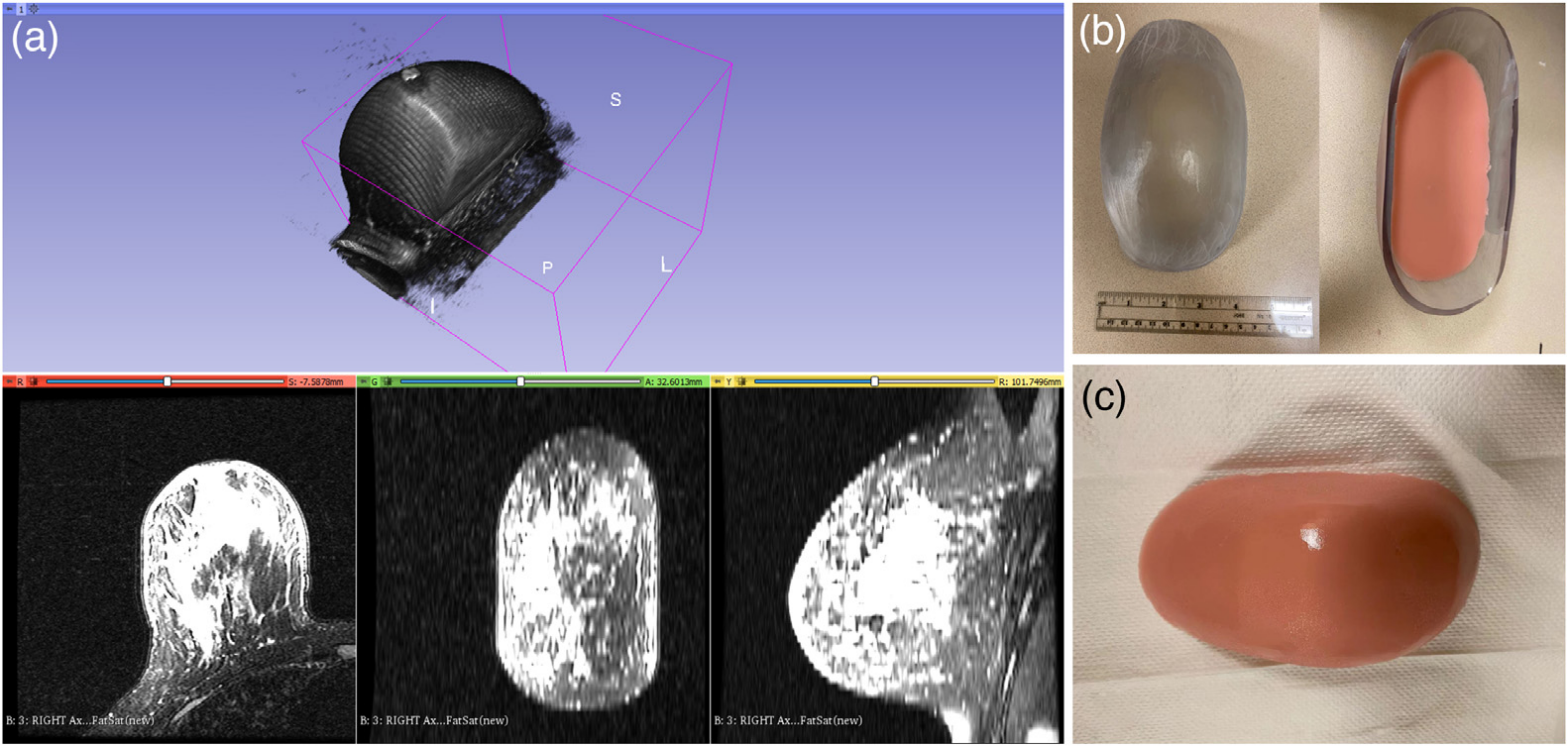
Breast-shaped phantom 3D-printed from MRI data. (a) 3D MRI of the subject’s breast. (b) 3D-printed mold, and (c) breast-shaped phantom after curing and removal from the mold.

To mimic a breast shape, 3D-printed molds were produced from MRI data. Figure 2 shows the process of creating breast phantoms of different shapes. First, breast MRI data (or desired tissue/organ) were transferred from the scanner into solid works (design software) where a design model was generated using 3D slicer (NA-MIC) to create a mold [see Fig. 2(a)]. Then, based on this file, a mold was fabricated from plastic materials with a 3D printer (Stratasys J55) [see Fig. 2(b)]. Finally, the phantom base solution with pigments (to generate the desired optical properties) was poured into the mold to form the phantom [see Fig. 2(c)]. The shape of the phantom reproduced the same breast geometry extracted from the MRI scans.

### 2.3 Construction of Phantoms with Inclusions

**Fig. 3:**
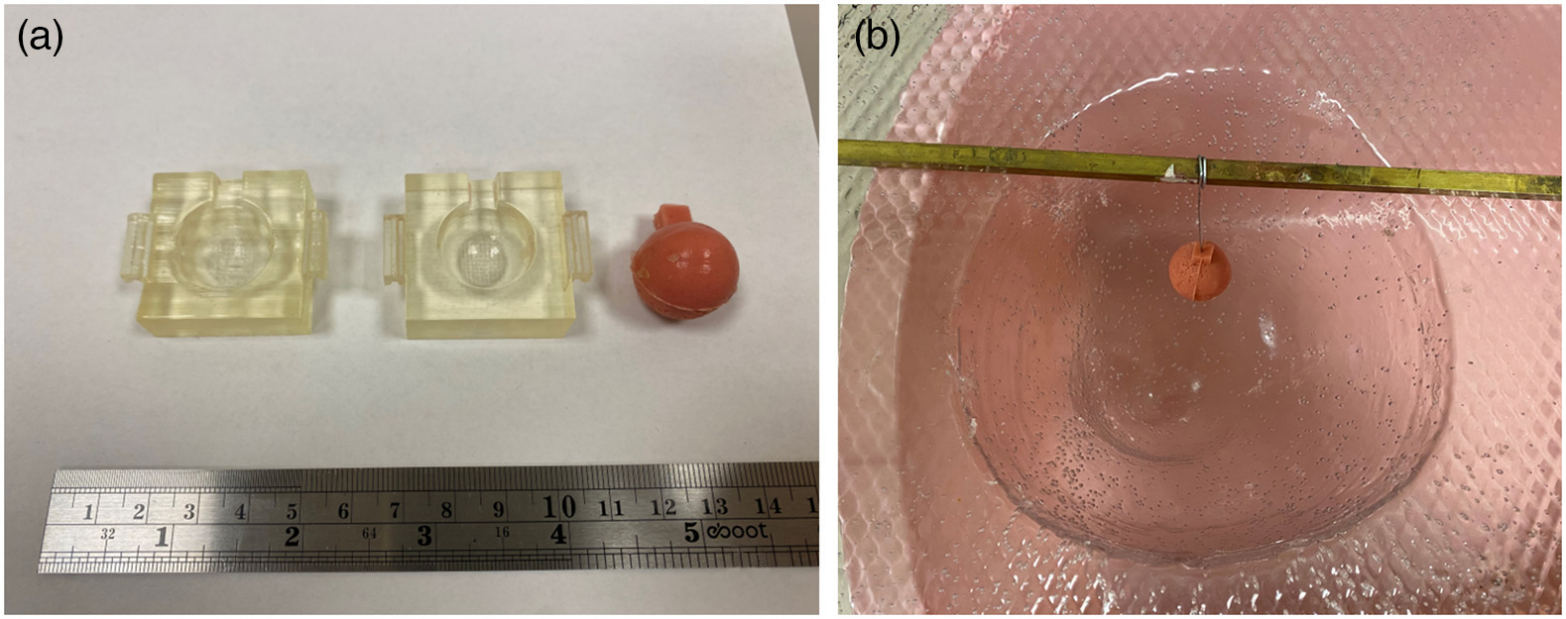
Phantom construction with an inclusion. (a) 25-mm inclusion with its mold; (b) inclusion was placed at the desired location within the mold before the base phantom solution was added.

To mimic a tumor surrounded by normal tissue for evaluating the performance of an imaging system, phantoms with inclusions were produced. As shown in Fig. 3, an inclusion having similar absorption ($\mu_{a}$) and scattering ($\mu_{s}^{\prime }$) coefficients of breast cancer (over the wavelength range of 600 to 850 nm) was constructed using a mold that represents the corresponding inclusion size and shape. In this study, a 25-mm spherical-shaped inclusion was made [Fig. 3(a)]. Then, the inclusion was placed at the desired location within the breast-shape of a mold by suspending it from a rigid thin metallic wire. After pouring the phantom base solution (already conjugated with the catalysts) into the mold and letting the material set, the thin metallic wire was pulled out, and a mark was made on the phantom surface to indicate the position of the inclusion.

### 2.4 Measuring Optical Properties

A diffuse optical spectroscopic imaging (DOSI) system^16^^,^^17^ was used to retrieve phantom $\mu_{a}$ and $\mu_{s}^{\prime }$ values at wavelengths 659, 691, 786, and 829 nm, and the spectrum was recorded over the wavelength range from 400 to 1300 nm.^16^ The average of three repeat measurements at different positions within the phantom was used to represent the final optical properties of the corresponding homogeneous phantoms.

### 2.5 Multimodal Imaging

Phantoms with inclusions were imaged with CT and MRI to evaluate their multimodal contrast. CT imaging was carried out on a Siemens SOMATOM Open Pro at 120 kVp. MR imaging occurred on a Siemens 3T Magnetom Prisma Fit scanner (Siemens). T1 and T2w STIR sequences with TR/TE = 5.43/2.46 ms and TR/TE = 2310/70 ms were utilized.^18^ Concurrent optical imaging data were acquired with T1 MRI, and the optical images were reconstructed with an MRI-guided near-infrared spectroscopic tomography (MRg-NIRST) reconstruction approach.^19^^,^^20^ To estimate the inclusion to background contrast, two circular regions of interest (ROIs) with diameters of 20 mm were picked in the inclusion and background, and the average values in each ROI were calculated. Contrast was defined as the ratio of the average value in inclusion to that in the background ROIs.

## 3 Results

To demonstrate that optical properties can be controlled by adjusting concentrations of the pink and white pigments, a group of phantoms was formed from 50 ml of base A and 5 ml of catalyst B base solution with different concentrations of pigments. White pigment concentrations were 0.00%, 0.55%, 0.73%, and 1.45%, respectively, and pink pigments were increased from 0.36% to 1.82% in increments of 0.364%.

**Fig. 4:**
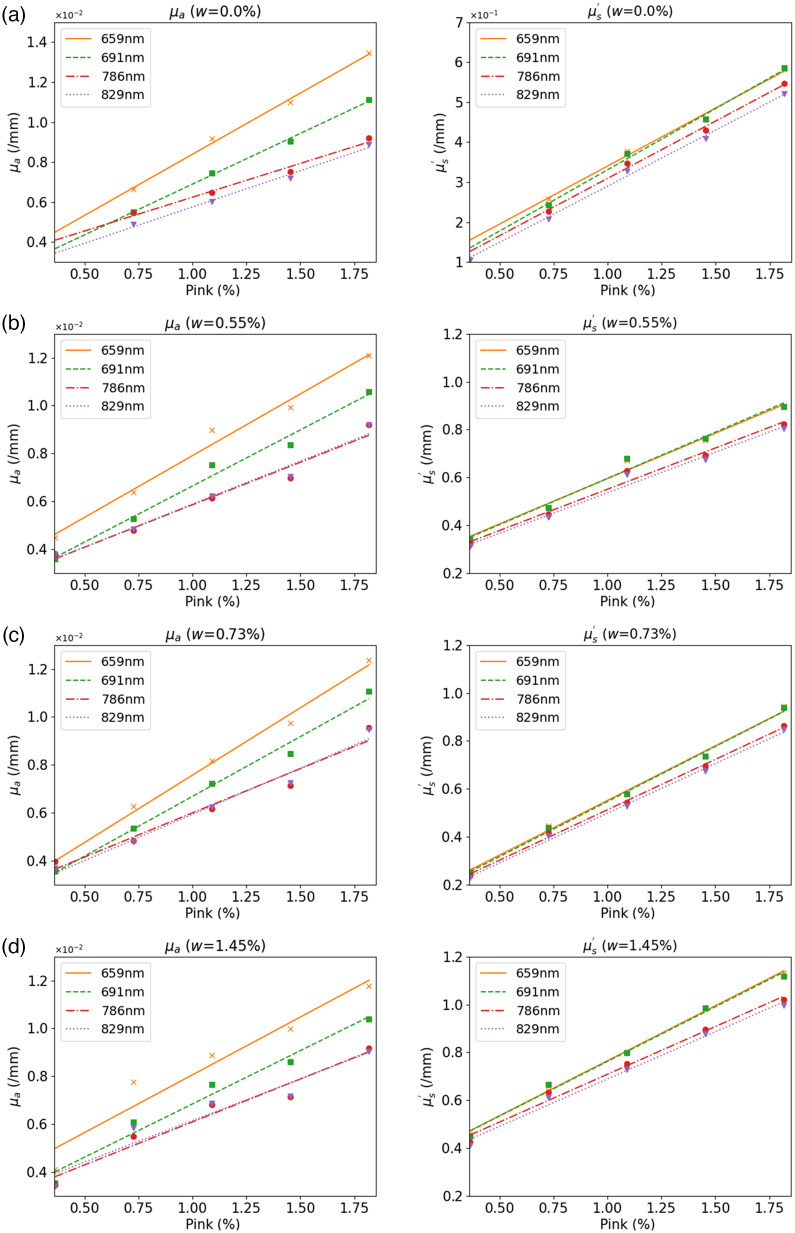
Retrieved values of $\mu_{a}$ (left) and $\mu_{s}^{\prime }$ (right) at four different NIR wavelengths. Concentrations of pink pigment changed from 0.36% to 1.82% in increments of 0.364%. Lines and dots in different colors show results at wavelengths of 659 (orange), 691, 785 (red), and 829 nm (purple). The concentrations of white pigment were (a) 0.00%, (b) 0.55%, (c) 0.73%, and (d) 1.45%.

Figure 4 shows $\mu_{a}$ and $\mu_{s}^{\prime }$ of these phantoms retrieved from DOSI measurements. $\mu_{a}$ and $\mu_{s}^{\prime }$ were plotted versus pink pigment concentration at wavelengths of 659 nm (orange), 691 nm (green), 786 nm (red), and 829 nm (purple). The concentrations of white pigment were 0.00% [Fig. 4(a)], 0.55% [Fig. 4(b)], 0.73% [Fig. 4(c)], and 1.45% [Fig. 4(d)]. $\mu_{a}$ and $\mu_{s}^{\prime }$ both increased linearly from 0.004 to $0.4\;{\mathrm{mm}}^{-1}$ to 0.012 and $1.2\;{\mathrm{mm}}^{-1}$ with different slopes, when concentrations of the pink pigment increased from 0.25% to 1.75%, at each wavelength. Linear regression coefficients, $R^{2}$, of $\mu_{a}\;$and $\mu_{s}^{\prime }$ were higher than 0.940 (average was 0.974) and 0.925 (average was 0.976), respectively, indicating excellent linearity in $\mu_{a}$ or $\mu_{s}^{\prime }$ with pink pigment concentrations. As shown in Figs. 4(b)–4(d), a similar linearity was found at the other white pigment concentrations.

**Fig. 5:**
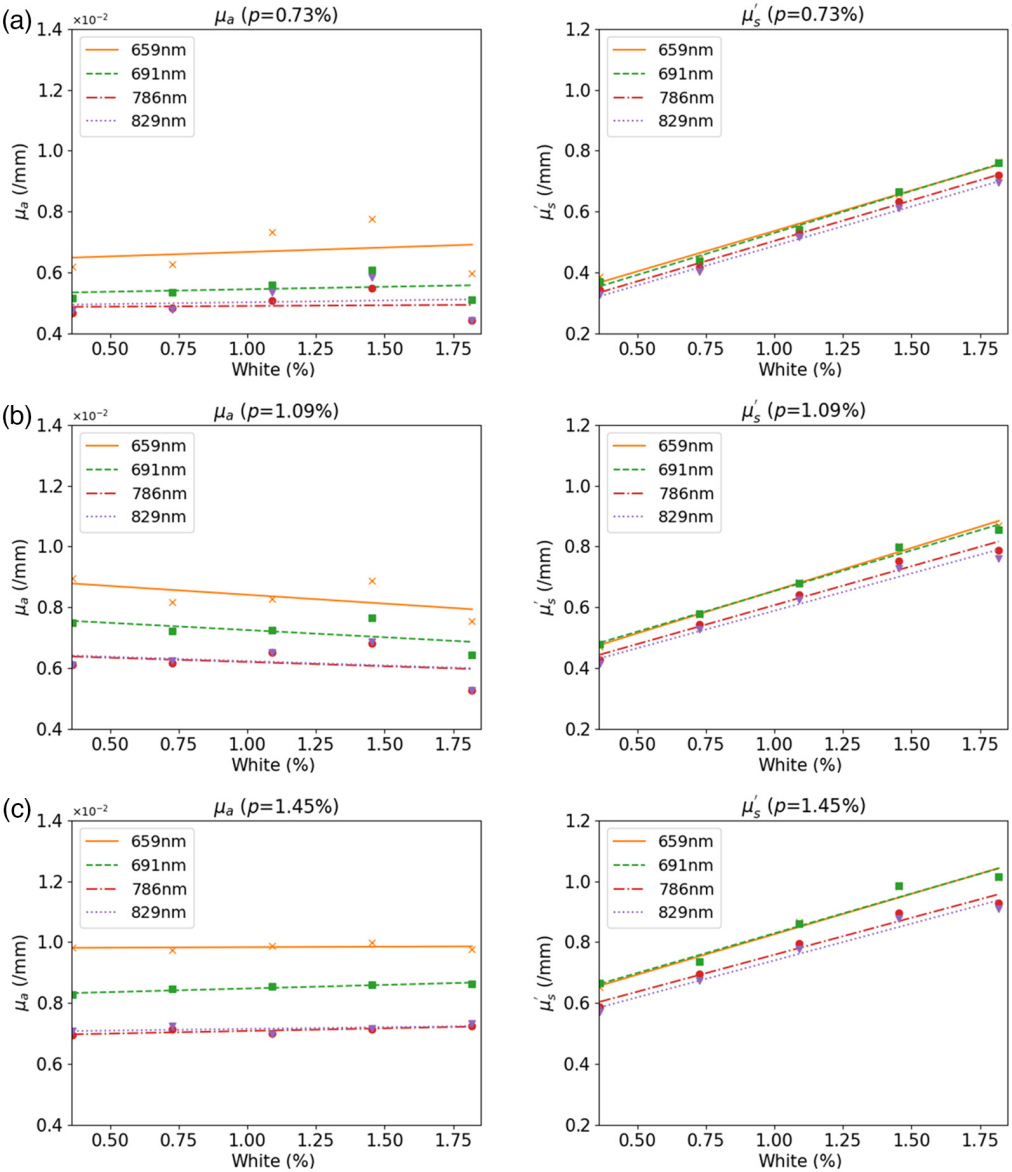
Retrieved values for $\mu_{a}$ (left) and $\mu_{s}^{\prime }$ (right) at four different NIR wavelengths are plotted when white pigment concentration changed from 0.36% to 1.82% in an increment of 0.364%. Lines and dots with different colors show results at wavelengths of 659 (orange), 691, 785 (red), and 829 nm (purple). Concentrations of pink pigment were (a) 0.73%, (b) 1.09%, and (c) 1.45%.

Figure 5 shows $\mu_{a}$ and $\mu_{s}^{\prime }$ when white pigment concentrations changed from 0.25% to 1.75% and pink pigment concentration was fixed at 0.73% [Fig. 5(a)], 1.09% [Fig. 5(b)], and 1.45% [Fig. 5(c)], respectively. We could not determine values for white pigment alone (pink pigment concentration = 0%) because its absorption was too low to be measured by the DOSI system.

Compared with the significant changes in $\mu_{a}$ from pink pigment concentration changes, $\mu_{a}$ was mostly constant at each wavelength, when white pigment concentration changed from 0.73% to 1.45%. Although the white pigment concentration affected $\mu_{s}^{\prime }$ linearly with $R^{2}$ higher than 0.969 (average is 0.983), $\mu_{s}^{\prime }$ changes were smaller than those caused by pink pigment concentration changes (0.4 to $0.8\;{\mathrm{mm}}^{-1}$) in this case.

Table 1 lists the linear fitting parameters ($k_{p,\mu a}$, $k_{p,\mu s\prime }$, $b_{p,\mu a}$ and $b_{p,\mu s\prime }$) of the pink pigment needed for the desired $\mu_{a}$ and $\mu_{s}^{\prime }$ at the wavelengths of 659, 691, 786, and 829 nm, when using the linear equations

$$\mu_{a}=(k_{p,\mu a}\times C_{\mathrm{pink}}+b_{p,\mu a})\times 10^{-3},$$

$$\mu_{s}^{\prime }=(k_{p,\mu s^{\prime }}\times C_{\mathrm{pink}}+b_{p,\mu s^{\prime }})\times 0.1.$$

**Table 1:** Parameters for estimating $\mu_{a}$ (${\mathrm{mm}}^{-1}$) and $\mu_{s}^{\prime }$ (${\mathrm{mm}}^{-1}$) versus pink pigment concentrations (%) at fixed white pigment concentrations.

White pigment	659 nm	691 nm	786 nm	829 nm	659 nm	691 nm	786 nm	829 nm
	$k_{p,\mu a}$				$b_{p,\mu a}$			
0.00%	6.02	5.07	3.38	3.63	2.28	1.83	2.87	2.12
0.55%	5.15	4.68	3.55	3.59	2.76	1.96	2.31	2.31
0.73%	5.62	4.98	3.68	3.86	1.96	1.71	2.32	2.08
1.45%	4.82	4.45	3.59	3.47	3.24	2.40	2.51	2.68
	$k_{p,\mu s\prime }$				$b_{p,\mu s\prime }$			
0.00%	2.90	3.06	2.86	2.81	4.99	2.87	2.27	0.95
0.55%	3.78	3.86	3.44	3.39	2.17	2.10	2.07	1.97
0.73%	4.55	4.59	4.20	4.14	9.67	8.82	9.30	8.47
1.45%	4.59	4.56	3.99	3.95	3.06	3.06	3.10	2.94

Table 2 lists the linear fitting parameters ($k_{w,\mu s\prime }$ and $b_{w,\mu s\prime }$) of white pigment concentration needed for the desired $\mu_{s}^{\prime }$ at wavelengths of 659, 691, 786, and 829 nm, when using the linear equation

$$\mu_{s}^{\prime }=(k_{w,\mu s\prime }\times C_{\mathrm{white}}+b_{w,\mu s\prime })\times 0.1.$$

**Table 2:** Parameters for estimating $\mu_{s}^{\prime }$ (${\mathrm{mm}}^{-1}$) versus white pigment concentrations (%) at fixed pink concentrations.

Pink pigment	659 nm	691 nm	786 nm	829 nm	659 nm	691 nm	786 nm	829 nm
	$k_{w,\mu s\prime }$				$b_{w,\mu s^{\prime }}$			
0.7%	2.65	2.76	2.66	2.59	2.70	2.55	2.39	2.29
1.1%	2.81	2.67	2.56	2.46	3.74	3.87	3.52	3.43
1.5%	2.65	2.60	2.43	2.42	5.61	5.69	5.16	4.98

**Fig. 6:**
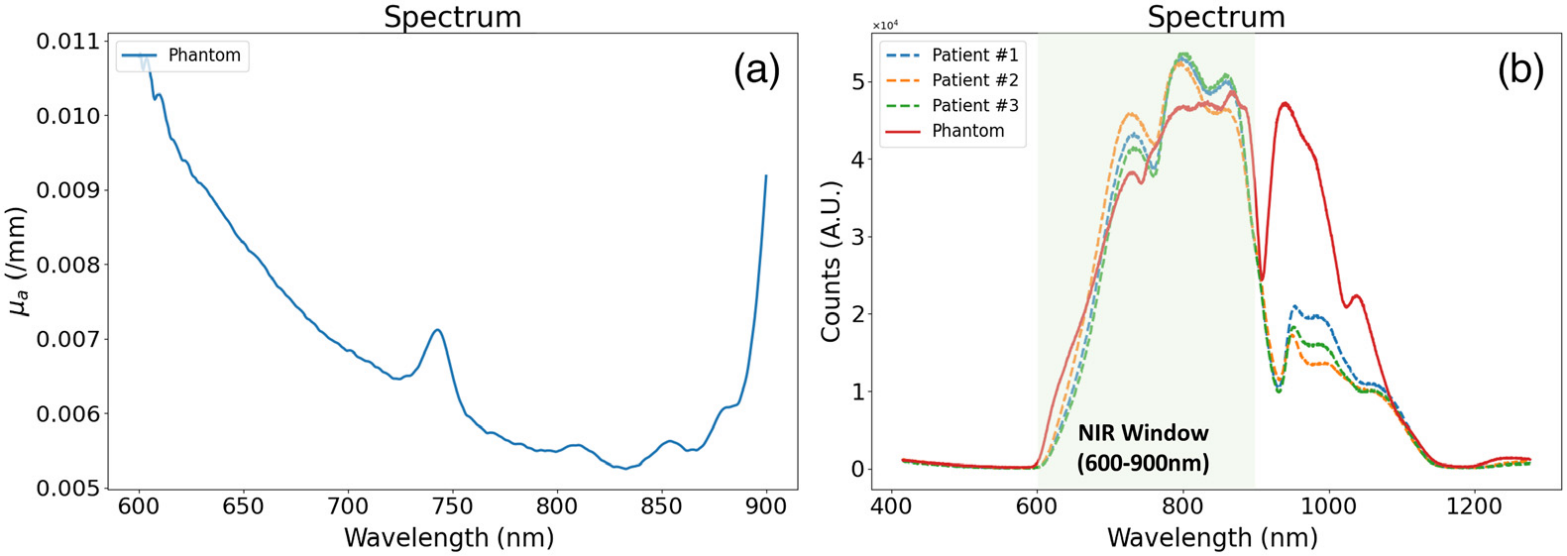
DOSI measured spectra. (a) $\mu_{a}$ spectrum of a phantom with pink pigment concentration of 1.0%; (b) attenuation spectra from a phantom and the breasts of three subjects. The red solid line represents the spectrum of a breast-shaped phantom, and three dashed lines of different colors represent the measured spectra from three subjects.

Figure 6(a) shows the $\mu_{a}$ spectrum of a phantom with pink pigment concentration of 1.0%, and Fig. 6(b) shows the attenuation spectra measured from a breast-shaped phantom as well as from the breasts of three subjects. Pink and white pigment concentrations in the breast-shaped phantom were 0.55% and 0.36%, respectively. Subjects #1, #2, and #3 were 66, 56, and 31 years old with each breast density of scatter (#1), heterogeneous dense (#2), and extremely dense (#3), respectively. Spectral features of the phantom are very similar to that in breast over the wavelength range from 600 to 900 nm.

To validate the phantoms’ long-term and short-term stability of optical properties, we measured the values of three randomly selected phantoms over 12 months. Pigment concentrations of these three phantoms were 0.55% white and 1.09% pink, 0.73% white and 0.73% pink, and 1.82% white and 0.73% pink, respectively. We found that the percentage changes (Δ%, standard deviation/mean value) in either $\mu_{a}$ or $\mu_{s}^{\prime }$ in these phantoms at different wavelengths were randomly changed within 2%.

To test the reproducibility of $\mu_{a}$ and $\mu_{s}^{\prime }$, three phantoms were constructed with different sizes and shapes but the same pigment concentrations (pink and white: 0.36% and 0.55%). As an example shown in Table 3, measured Δ% of $\mu_{a}$ and $\mu_{s}^{\prime }$ was <1.5% and 2%, respectively, at each wavelength. We did the same test on the phantoms with the different pigment concentrations, and Δ% of $\mu_{a}$ and $\mu_{s}^{\prime }$ was in the same range.

**Table 3:** Retrieved $\mu_{a}$ and $\mu_{s}^{\prime }$ in three phantoms with the same pigment concentrations.

	Wavelength (nm)	Cylinder-shape #1	Cylinder-shape #2	Breast-shape
$\mu_{a}$ (${\mathrm{mm}}^{-1}$)	659	0.0064	0.0065	0.0064
	691	0.0053	0.0053	0.0052
	786	0.0048	0.0048	0.0047
	829	0.0048	0.0049	0.0048
$\mu_{s}^{\prime }$ (${\mathrm{mm}}^{-1}$)	659	0.48	0.47	0.48
	691	0.47	0.47	0.47
	786	0.45	0.45	0.45
	829	0.43	0.44	0.44

**Fig. 7:**
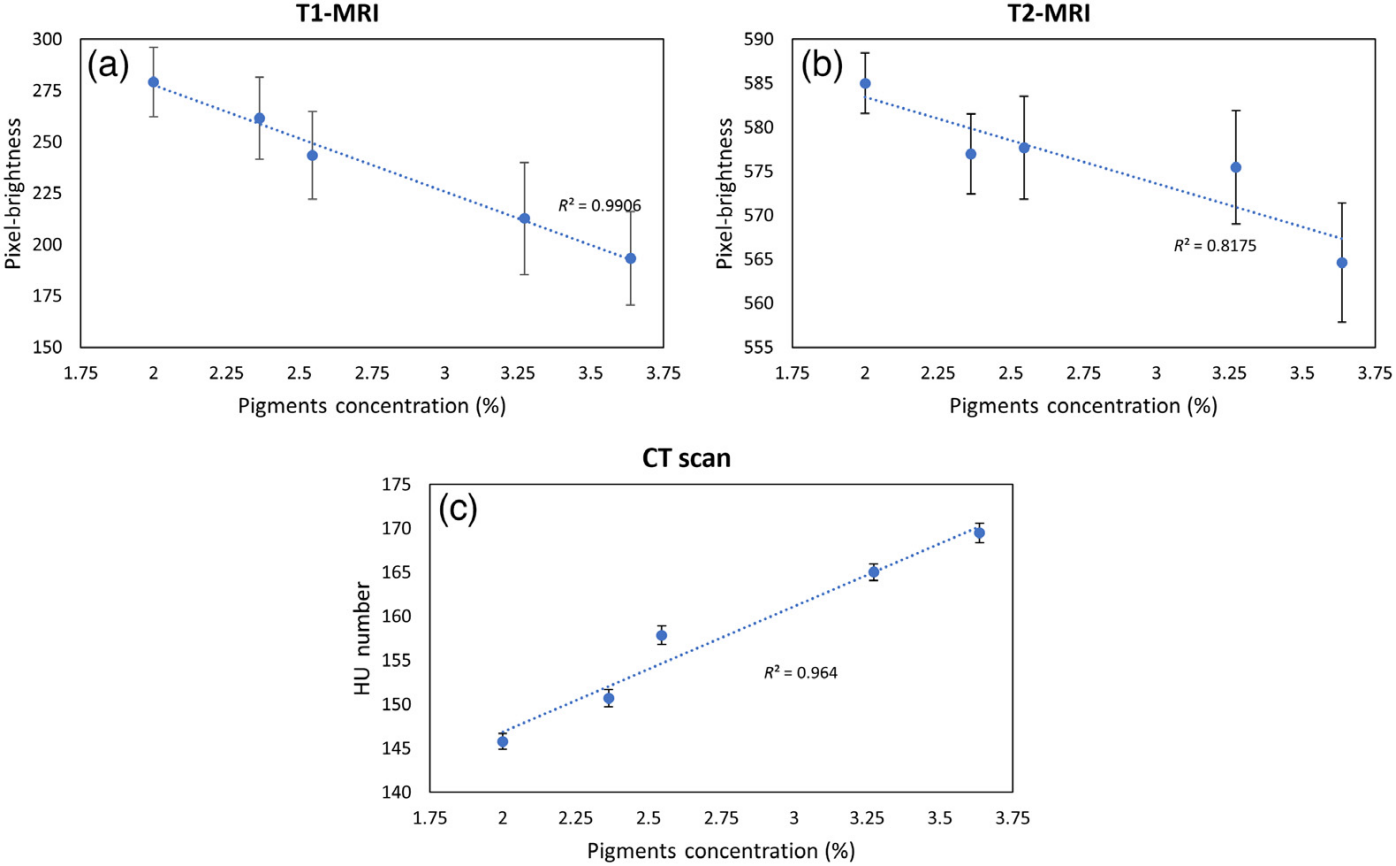
Pixel brightness in (a) T1 and (b) T2 MRI, as well as (c) CT numbers when imaged five phantoms with different pigment concentrations. The error bars refer to the standard deviation of the pixel brightness or CT numbers of the corresponding phantoms. Pigment concentrations (pink/white) of the five phantoms were 2.0% (0.18%/ 1.82%), 2.36% (0.55%/1.82%), 2.54% (1.09%/ 1.45%), 3.27% (1.82% /1.45%), and 3.64% (1.82%/1.82%).

To demonstrate that this new type of phantom can be used for validating and calibrating the multimodality imaging systems, five phantoms with different pigment concentrations were imaged with MR and CT. Although phantom optical properties were affected separately by either pink or white pigment concentrations, we found that the CT or MRI brightness was changed by the total pigment concentration regardless of whether it was white or pink. Figure 7 shows the measured pixel brightness in T1 and T2 MRI, as well as the CT number of these phantoms when the total pigment concentration was changed. The error bars refer to the standard deviation of the pixel brightness or CT numbers of the corresponding phantoms. The pigment concentrations (pink/white) of these five phantoms were 2.0% (0.18%/ 1.82%), 2.36% (0.55%/1.82%), 2.54% (1.09%/1.45%), 3.27% (1.82%/1.45%), and 3.64% (1.82%/1.82%). The brightness of both T1 and T2 MRI decreased, whereas CT numbers increased linearly with increasing pigment concentration, and the linear regression coefficients, $R^{2}$, were 0.99, 0.82, and 0.96.

**Fig. 8:**
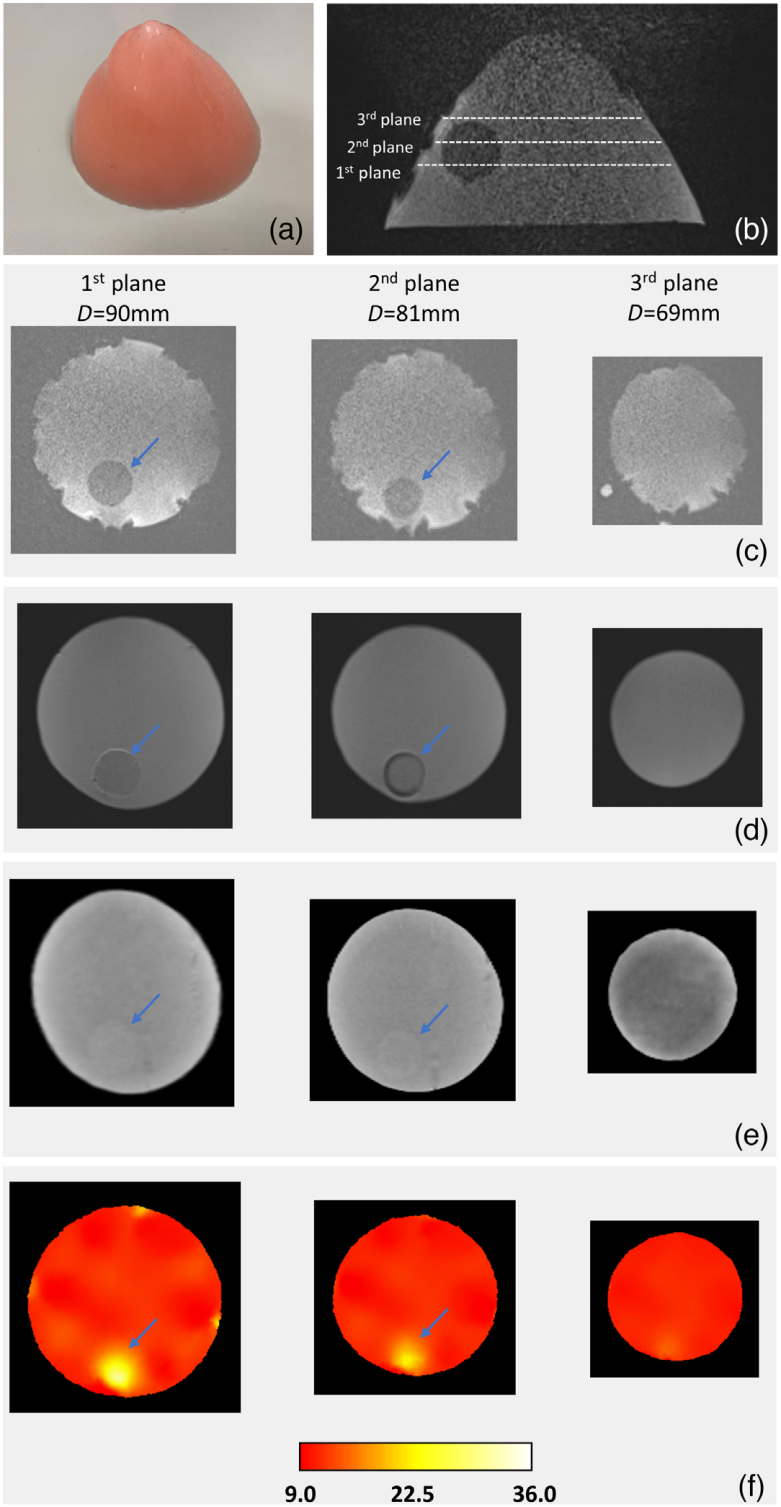
Photos and images of a breast-shaped MRI-NIRST multimodality heterogeneous soft gel phantom with a 25-mm-diameter spherical inclusion (identified by arrow). (a) Photo of a breast shape phantom; (b) axial view of the phantom in T1-MRI. Planes were determined by fiducials, which indicate optical detector layers for NIRST imaging; (c) T1-MR coronal view of first (left), second (middle), and third (right) layers with plane diameters of 90, 81, and 69 mm, respectively. MRI contrast of inclusion to background was ∼1∶1.5; (d) T2-MRI coronal images of these planes. Contrast of inclusion to background was ∼1∶1.2; (e) CT images of these planes. Contrast of inclusion to background was ∼1.1∶1, and ΔHU was ∼14; and (f) reconstructed HbT images of these planes by T1-MRI-guided NIRST reconstruction. Inclusion to background HbT contrast was ∼3∶1.

Figure 8 shows photographs and images of a heterogeneous soft gel phantom imaged by MRI, CT, and NIRST. A breast-shaped heterogeneous phantom [Fig. 8(a)] was fabricated and imaged with an MRI-NIRST and a CT system. The concentrations of the pigments of this phantom were 0.55% pink and 1.1% white, respectively. A 25-mm spherical-shaped inclusion with 1.1% pink and 2.2% white pigments was placed inside the phantom. The contrast of total hemoglobin in inclusion to that in the base phantom was 3.4:1; it was estimated through the spectra fitting using $\mu_{a}$ calculated from the pink and white pigment concentrations and the extinction coefficients^21^ of oxy-, deoxyhemoglobin, and water at the wavelengths of 661, 761, 785, and 826 nm. Figure 8(b) shows an axial view of the phantom in T1-MRI. Planes were determined by the fiducials that correspond to the optical detector planes for NIRST imaging.

The T1-MR coronal views are shown in Fig. 8(c), with the plane diameters of the first (left), second (middle), and third (right) optical detector planes of 90, 81, and 69 mm, respectively. Inclusion edges are sharp and clear in the MR and CT images [Fig. 8(c)–8(e)], and the diameters of the inclusion are 24 and 21 mm in first (left) and second (middle) planes, whereas no inclusion appears in third (right) plane. T1 and T2 MRI contrasts of the inclusion to the background were 1:1.5 [Fig. 8(c)] and 1:1.2 [Fig. 8(d)], respectively, whereas the CT contrast was 1:1.1 [Fig. 8(e)]. ΔHU of ∼14 in our CT images was comparable to that of the fibroglandular to breast cancer contrast (∼20).^22^ Total hemoglobin (HbT) images in Fig. 8(f) were recovered through an MRg-NIRST image reconstruction^23^ based on measured amplitude data and $\mu_{s}^{\prime }$ estimated from the pink and white pigment concentrations of the base phantom at four wavelengths. Inclusion diameters estimated from NIRS images in first (left) and second (middle) were 21 and 18 mm, respectively. These values are similar to that shown in MRI and CT images [Figs. 8(c)–8(e)]. The 12% to 16% inclusion size differences obtained in MRI/CT and NIRST images are imaging errors resulting from the relatively low NIRST spatial resolution. The inclusion to the background contrast was about 3:1, which is similar to that of 3.4:1 estimated from the spectra fitting based on $\mu_{a}$ estimated from the pigment concentration.

## 4 Discussion

Tissue-mimicking phantoms with spectral properties similar to soft tissues have been investigated in the past, and many useful models have been created.^2^ Characteristic tissue phantoms have been proven to be indispensable tools for evaluating optical imaging modalities, such as near-infrared spectral tomography, photodynamic therapy,^24^ luminescence imaging,^25^ fluorescence molecular imaging,^26^ and optical coherence tomography.^10^^,^^27^ Table 4 summarizes the preparation time, expected longevity, advantages, and drawbacks of the solid phantoms developed in the past and the relative to the soft gel phantom described here.

**Table 4:** Comparison of major tissue-mimicking phantoms.

Types	Prep. time	Longevity	Advantages	Drawbacks
Gelatin^28^	3 h	<1 day	Blood and organics as absorbers	Very temporary use
			MRI/CT contrast	Need accurate temp. control
Resin^29^	24 h	>1 week	Stable optical properties	No control of elastic properties
				Air bubbles
RTV^30^	5 to 6 days	>1 week	Mimic tissue elastic properties	Simple structures
				Difficult to control $\mu_{s}^{\prime }$
3D printed^12^^,^^13^	24 h	>1 week	Complicated structures	Narrow-band optical properties
Soft gel	1 h	>1 week	Short creation time	Simple structures
			Room temperature	
			Mimics elasticity	
			MRI/CT contrast	

Gelatin phantoms mainly use water and agarose/gelatin powder as the base materials and blood/intralipid as the absorbers/scatters. The most attractive feature of gelatin phantoms is their compatibility with blood and/or fluorescence agents for mimicking optical spectra and fluorescence properties of soft tissues. However, gelatin phantoms are fragile, and their lifetime is <1 day under room temperature conditions. In addition, the fabrication process takes about 3 h, and the temperature must be controlled accurately from 80°C to 4°C using a microwave oven or a refrigerator at different time points during construction. Resin phantoms are formed from resin, hardener, ink, and titanium dioxide (${\mathrm{TiO}}_{2}$). They can last for many years with stable optical properties; however, production requires the phantom to be degassed in a vacuum to prevent bubbles from forming during chemical reactions of the resin with the hardener, and the curing procedure typically takes around 24 h. Indeed, because the ink is used for mimicking the tissue absorber, the optical properties can only be matched at a particular wavelength. Elastic properties of RTV-based silicone phantoms can be adjusted to mimic a particular target tissue; however, the curing procedure of some RTV phantoms can take 5 to 6 days if the elasticity is modulated by varying the hardener concentration.^31^

In recent studies, 3D-printed phantoms showed the potential to improve medical imaging by providing complex structures that resemble tissue structures more closely. However, the materials that can be used for 3D printing are limited, and accurately adjusting optical properties, especially scattering properties, of the materials to mimic specific tissues over a relatively wide wavelength range is difficult.^13^

In contrast, the fabrication of the soft gel phantom described here is simple and flexible. The entire process can be accomplished at room temperature and within an hour. The shape of the phantom can be molded, making construction of complex layered structures possible by adding layers repeatedly through different molds. Optical properties of these phantoms were very stable over months so they can be used in long-term image quality control studies.

As shown in Figs. 4 and 5, $\mu_{a}$ and $\mu_{s}^{\prime }$ at a certain wavelength can be controlled by linear adjustments of the concentrations of pink and/or white pigments using parameters listed in Tables 1 and 2. Because the white pigment only changes $\mu_{s}^{\prime }$, we first estimated the pink pigment concentration at a fixed concentration of white pigment (empirically setting white concentration to 0.73%) to match $\mu_{a}$ and then adjusted the white pigment concentration to match $\mu_{s}^{\prime }$.

To evaluate optical spectra, we selected data from three patients collected with the DOSI system. Although the attenuation spectra of the patients [dashed lines in Fig. 6(b)] were slightly different from each other, the overall spectral trends were the same, (i.e., gradual increase, then plateau with some fluctuation, and final drop over the wavelength ranges from 600 to 725, 725 to 875, and 875 to 900 nm, respectively). Compared with these breast tissue spectra, the overall trend of the attenuation spectrum of the soft gel phantom [red solid lines in Fig. 6(b)] was similar at wavelengths shorter than 900 nm. Combined with the results shown in Fig. 8 that phantoms can represent total hemoglobin using the extinction coefficients of oxy- and deoxyhemoglobin and water, our phantoms are appropriate for mimicking functional biological properties of soft tissue for molecular spectroscopy and imaging.

Although the inclusion to background contrast in a heterogeneous phantom has not been evaluated directly over time, we expect the contrast not to change during 12 months because the optical properties in the homogeneous phantoms did not change, and the pigments cannot diffuse between the inclusion and background over time once the inclusion and background phantom components are solidified.

As shown in Figs. 7 and 8, another unique feature of the soft gel phantom is the different concentrations of pink and white pigments contributed to contrast in CT and MRI. Relative to gelatin phantoms in which secondary materials are required to generate MRI or CT contrast (and these secondary agents often diffuse across boundaries with different optical properties under room temperature), the boundaries in our new soft gel phantoms with different optical properties are clear and sharp in MRI or CT—a feature that improves quality control in multimodality imaging studies.

In general, T1 MRI and CT contrasts in breast cancer (relative to surrounding normal breast tissue) are ∼1.75∶1 (positive) and ∼1.2∶1 (positive, ΔHU of ~20), respectively. The HbT inclusion to the background contrast in the phantom obtained by NIRST was 3, which is similar to that of actual breast cancer [see Fig. 8(f)], whereas the T1 MRI and CT phantom contrasts were 1:1.5 (negative) and 1.1:1 (positive, ΔHU of ∼14), which are 14% and 8% lower than the breast cancer to the normal tissue contrasts observed in practice, respectively. Nonetheless, the phantom inclusion to background contrast is sufficiently clear to indicate the inclusion location and size for co-registration of optical to MR or CT images.

We also imaged this phantom with the DOSI system.^16^^,^^17^ The inclusion was detectable and the background (normal tissue) HbT was ∼13  μM, which is slightly higher than the value obtained by NIRST and the corresponding theoretical calculation [Fig. 8(f)]. However, HbT of the inclusion was ∼15  μM, leading to an inclusion to background contrast of ∼1.15, which is much lower than the calculated contrast from the pink dye concentrations in the inclusion versus background (=3:1). Reasons for the lower contrast include (1) nonuniform probe contact with the curved surface of the breast phantom, which violated the semi-infinite boundary condition assumption required of DOSI, and (2) the inclusion depth, which affected the measurement results because DOSI uses the reflectance mode to form the tomographic image.

One drawback of the new soft gel phantom is that the base material is water insoluble, which limits the use of these phantoms in applications that require liquid contrast or fluorescence agents and/or other pigments that mimic optical properties in other wavelength ranges. Another minor drawback is the high reflectivity of the phantom surface, in which case, achieving skin-like reflectance needs specially designed and frosted inner mold surfaces.

## 5 Conclusion

A new type of optical tissue-mimicking phantom has been developed for optical and multimodality imaging. Phantom construction takes less than an hour and does not involve heating, cooling, or a vacuum. Phantom absorption and scattering properties can be controlled easily at any desired wavelength over the red and NIR wavelength range by adjusting the concentrations of white and pink pigments. The phantom material components are readily available commercially, and they provide stability for over 12 months. In addition, the pink and white pigments demonstrate the contrast in both CT and MRI as a function of concentration; hence, these phantoms can be used in multimodality imaging studies without introducing secondary contrast agents for CT or MRI.
